# An Insight on Multicentric Signaling of Angiotensin II in Cardiovascular system: A Recent Update

**DOI:** 10.3389/fphar.2021.734917

**Published:** 2021-08-20

**Authors:** Kanika Verma, Malvika Pant, Sarvesh Paliwal, Jaya Dwivedi, Swapnil Sharma

**Affiliations:** ^1^ Department of Pharmacy, Banasthali Vidyapith, Banasthali, India; ^2^ Department of Chemistry, Banasthali Vidyapith, Banasthali, India

**Keywords:** angiotensin II, stem cell, biomarkers, hypertrophic markers, cardiac gene regulation, signaling

## Abstract

The multifaceted nature of the renin-angiotensin system (RAS) makes it versatile due to its involvement in pathogenesis of the cardiovascular disease. Angiotensin II (Ang II), a multifaceted member of RAS family is known to have various potential effects. The knowledge of this peptide has immensely ameliorated after meticulous research for decades. Several studies have evidenced angiotensin I receptor (AT_1_ R) to mediate the majority Ang II-regulated functions in the system. Functional crosstalk between AT_1_ R mediated signal transduction cascades and other signaling pathways has been recognized. The review will provide an up-to-date information and recent discoveries involved in Ang II receptor signal transduction and their functional significance in the cardiovascular system for potential translation in therapeutics. Moreover, the review also focuses on the role of stem cell-based therapies in the cardiovascular system.

## Introduction

In the last decades, researchers have successfully unraveled key functions and mediators of the renin-angiotensin system (RAS). Ubiquitously available RAS plays numerous physiological roles including regulation of blood pressure, fluid volume, vascular wall integrity, cell growth, cardiac output, and vascular tone in the body (Forrester et al., 2018). RAS is also involved in maintaining cardiovascular homeostasis, a network of intracellular signaling pathways, and various processes through endocrine, paracrine, and autocrine mechanisms (Bussard and Buss, 2018). Regardless of complexities associated with its movement from the local system occurring virtually in each organ to the hormonal system existing in circulation, the active end product is still Angiotensin II (Ang II) (Colafella and Danser, 2017).

Historically, in 1898, renin was discovered as a pressor compound within the extracts of the renal cortex of rabbits by Robert Tigerstedt. Their work was renewed in 1934, when Henry Goldblatt demonstrated induction of chronic hypertension by constriction of renal arteries in a dog with silver clamps. In continuation to this, Page and Helmer and Braun-Menéndez et al., discovered angiotensin as another compound from renal secretion bearing quick pressor response. These studies focused on the involvement of Ang II in physiological and pathophysiological functions. Besides, RAS inhibiting agents have shown promising benefits in the management of end-organ damage, ischemia, atherosclerosis, and cardiovascular-related disease (Nehme, 2019). A timeline of key historical findings associated with the study and discovery of Ang II associated with RAS is shown in Table 1 (Burton et al., 1985; Gibbons, 1998; Basso and Terragno, 2001; Andrea et al., 2006; Atlas, 2007; Skrbic and Igic, 2009; Benigni et al., 2010).

**TABLE 1:** Glimpse of the historical development of RAS.

Discoverer (Year)	Development in RAS
Richard Bright (1836)	Related hypertrophy to an increased resistance to blood flow in the small vessels due to the altered condition of the blood
George Johnson (1868)	The pathology behind left ventricular hypertrophy
F.A. Mahomed (1872)	➢ Described high blood pressure using a primitive sphygmograph
	➢ Linked left ventricular hypertrophy to hypertension due to nephritis
	➢ Presence of high blood pressure in patients without renal disease
Riva Rocci (1896)	Introduced first indirect sphygmomanometer to measure arterial pressure in humans
Tigerstedt and his assistant Bergman (1898)	➢ Analyzed and discovered the presence of a pressor compound in the renal tissue ‘Renin’
	➢ Explained association between renal disease and cardiac hypertrophy
Korotkoff (1905)	Defined the cardiac sounds
Goldblatt et al. (1934)	➢ Linked ischemic characteristic of renal disease with hypertension
	➢ Induced experimental hypertension in a dog by partial constriction of a renal artery using a silver clip
	➢ Proposed the existence of a humoral mechanism
Irvine. H. Page heading Indianapolis group (1940)	Discovered renin as an inactive enzyme, activated by plasma protein compound renin activator and they named angiotensin
Edward Braun Menendez heading Argentine group (1940)	➢ Described renin as an enzyme similar to papain, which could act on a protein present in the plasma and named it hypertensin
	➢ Braun-Menendez and Page then agreed to name this new substance angiotensin
Argentine group (1943)	Research performed on the RAS by Argentine group were published in a book
Skegg’s et al. (1956)	➢ Revealed that angiotensin-converting enzyme (ACE), an endothelial bound enzyme in lungs, plasma, and also in the vascular bed of brain, heart, and kidney can convert angiotensin I to angiotensin II
	➢ Highlighted the amino acid sequence for angiotensin II
	➢ Angiotensin was first isolated in pure form from the reaction product of rabbit renin and beef blood
Braun Menéndez (1958)	➢ Renin substrate was named angiotensinogen
	➢ Enzymes that metabolize the peptide were termed angiotensinases

In view of traditional applications, investigators are making a consistent effort to explore the associated pharmacological effects of Ang II. Unfortunately, it is hoped that the next 100 years of research into RAS will uncover hitherto unimaginable therapeutic opportunities (Ferrario, 2006). The review will provide recent findings on Ang II receptor signal transduction and its functional significance in the cardiovascular system. In addition to this, the review also focuses on the applications of stem cell-based therapies in the cardiovascular system. The majority of pathophysiological conditions including hypertension and cardiac remodeling of Ang II are mediated by AT_1_ R, which makes specific signaling pathways much clearer. In light of these facts the purpose of the present review is to provide newer insights in future research with an instinct that it will help emerging novel strategies to establish Ang II as a promising therapeutic candidate in translational research in the near future.

## Method: Exclusion and Inclusion Criteria

The articles, written in English, published from 1985 to 2020, were exploited for gathering all relevant information of Ang II related articles from search databases namely, Science Direct, Medline/PubMed, Google Scholar, and other sources. Various databases were used to identify peer-reviewed papers dealing with the review theme of angiotensin-induced cardiovascular issues. A pilot review of literature assisted in identifying search terms that were used to categorize articles through a standardized and systematic process. The strings/words used for search purposes were as follows: “angiotensin”, “induced”, “receptor”, “signaling”, “disease”, “mediators”, “animal model”, “biomarkers”, “hypertrophic markers”, “cardiac genes”, “stem cells and others”.

## Angiotensin II Receptors and Signaling Pathways

**FIGURE 1:**
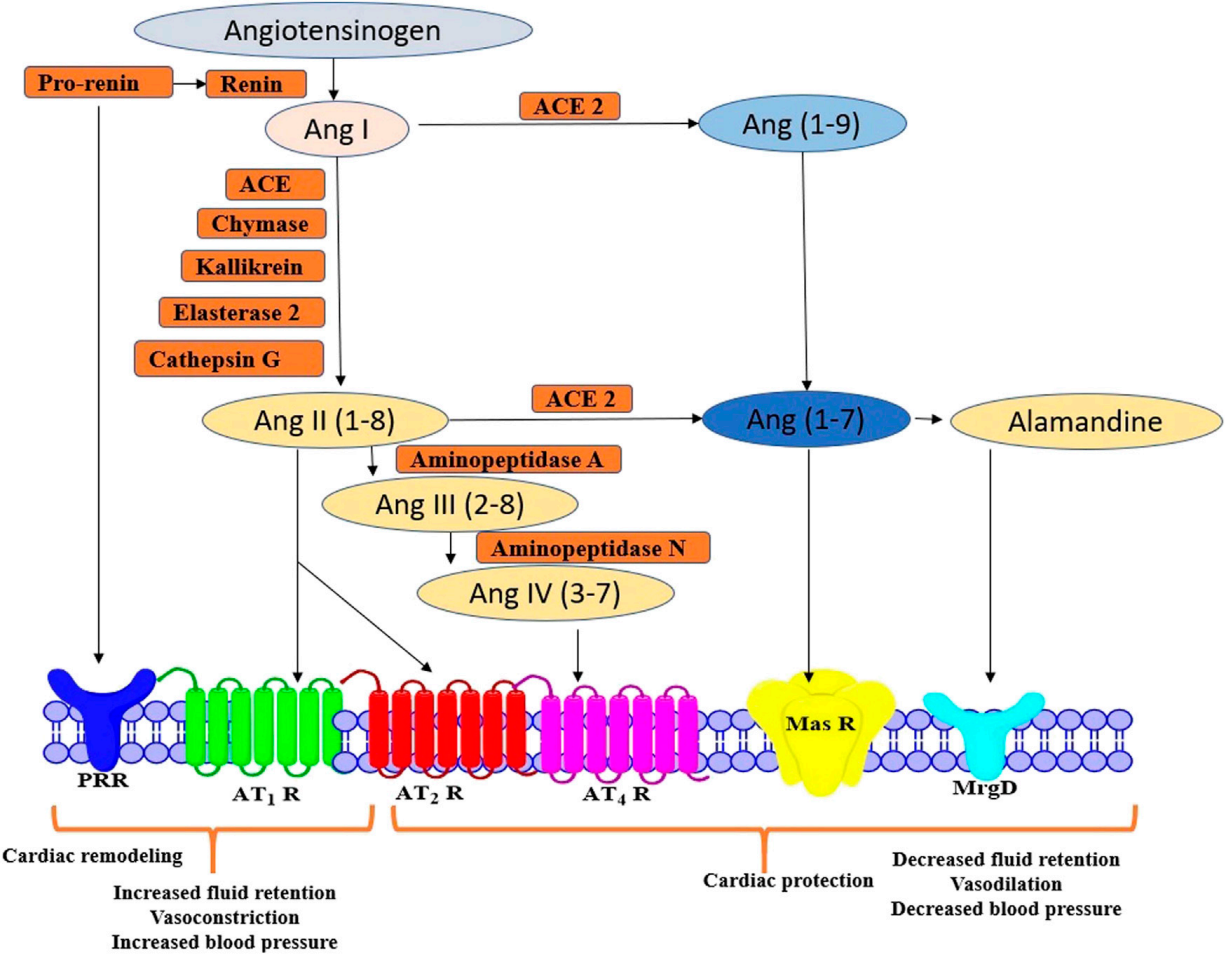
Schematic representation of Ang II peptides and receptors in RAS signalling pathway AT_1_ R, Angiotensin II Type 1 Receptor; AT_2_ R, Angiotensin II Type 2 Receptor; ANG II, angiotensin II; Ang-(1–7), Angiotensin-(1–7); Ang-(1–9), Angiotensin-(1–9); ACE2, Angiotensin-converting Enzyme 2; Mas R, Mitochondrial assembly protein Receptor.

RAS involves different peptides with opposing biological effects. To sum up, the pro-inflammatory, pro-proliferative, and vasoconstrictive molecules are Ang II, AT1 R, and angiotensin-converting enzyme (ACE). Contrarily, AT2 R, ACE2, Ang (1–7), MrgD and MasR, exerts cardio-protective effects. In brief, angiotensinogen produced from the liver is converted into Ang I and Ang II via renin, esterase-2, cathepsin G, kallikrein, chymase, and angiotensin-converting enzyme. Ubiquitous actions of Ang II can be attributed to activation of several signal transduction pathways modulated by receptors including AT_1_ R and AT_2_ R to initiate RAS or further get cleaved into peptides namely, Ang IV, Ang (1–7), and alamandine, which express their effects via AT4 R, Mas R and MrgD, respectively (Adamcova et al., 2021; Matsubara, 1998). Interestingly, administration of Ang (1–7) was evidenced to provide a protective effect during chronic infusion of Ang II in rats (Grobe et al., 2007). However, the pharmacology of AT_3_ R and AT_4_ R has not been categorized fully and hence they are not definitively classified under mammalian Ang receptors (Figure 1) (Touyz and Berry, 2002). Based on several investigations, AT_1_ R and AT_2_ R have been evidenced to be associated with the majority of Ang II mediated signaling pathways (Kawai et al., 2017). AT_1_ R is clearly different from AT_2_ R in signalling mechanisms, tissue-specific expressions, and molecular weight. AT_2_ R may also counter-regulate functions mediated via AT1 R. However, the signaling mechanisms of AT_2_ R are still speculative compared with those of AT_1_ R. Moreover, most of the classic cardiovascular effects of Ang II are conveyed by AT_1_ R, including, vasoconstriction, hyperplasia, sodium retention, vascular cell hypertrophy, myocardial fibrosis, arterial wall thickening, aggravation of inflammatory responses, and stimulation of ROS.

**FIGURE 2:**
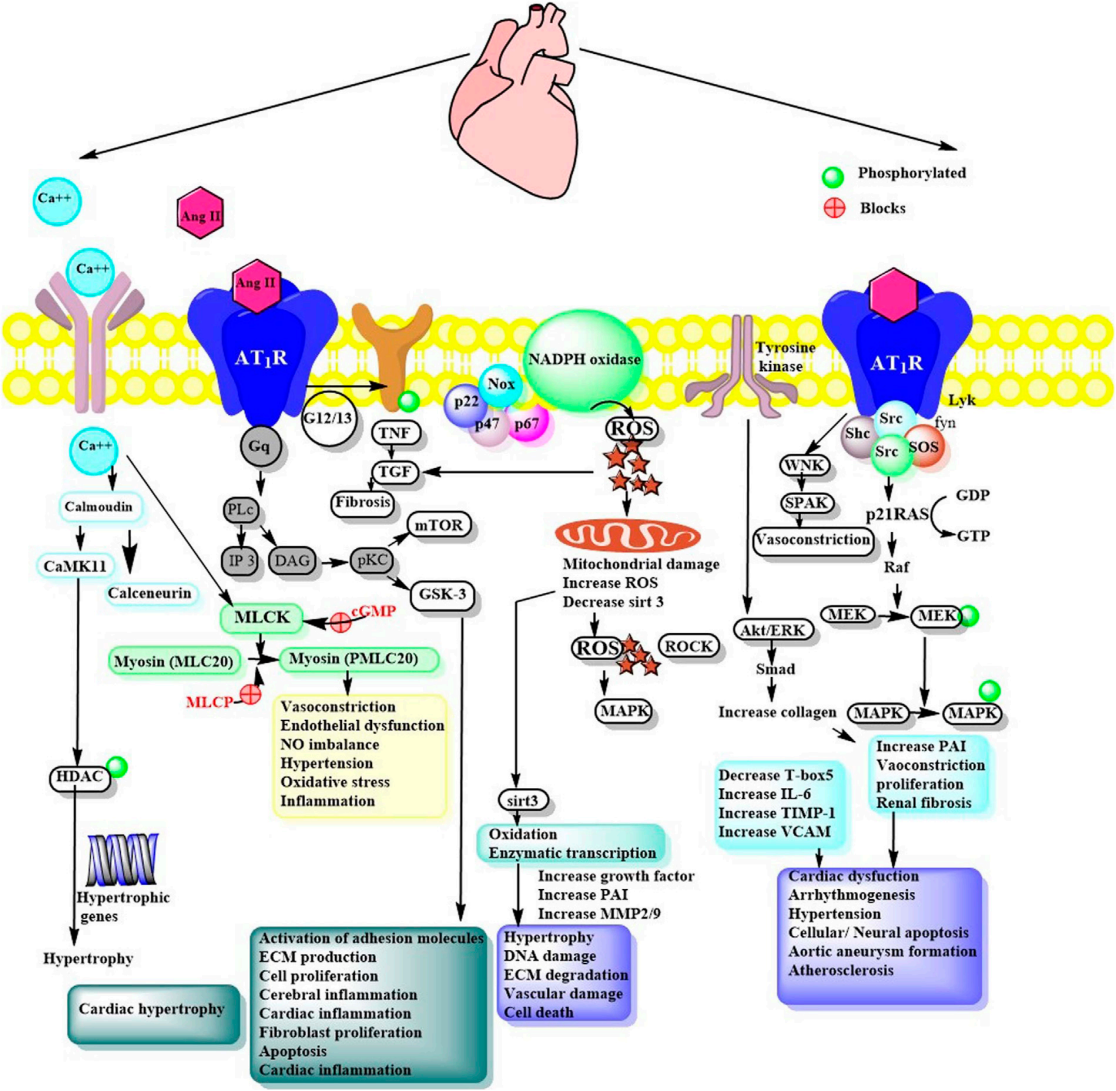
Ang II signaling via AT_1_R mediated pathways. Ang II, Angiotensin II; Ca^++^, Calcium; CaMKII, Ca2+/calmodulin-dependent protein kinase II; NFAT, Nuclear factor of activated T-cells; HDAC, Histone deacetylases; PLc, phospholipase C; IP3, Ionositol triphosphate; DAG, Diacyl glycerol; pKc, Protein kinase; CaMKII, Calcium/calmodulin- dependent protein kinase-II; NFAT, Nuclear factor of activated T-cells; MLCK, Myosin light chain kinase; MAPK, Mitogen activated protein kinase; PAI, Plasminogen activator inhibitor; NFKB, Nuclear factor-kB; MMP, Matrix metalloproteinase; TIMP, Tissue inhibitor metalloproteinase; VCAM, Vascular cell adhesion molecule; PVN, Paraventricular nucleus; ECM, Extracellular matrix; HDAC, Histone deacetylase; GTP, GDP, Nucleotides; Akt, Protein kinase B; cGMP, Guanosine 3’,5’ cyclic monophosphate; ROS, Reactive oxygen species; Grb2, Growth factor receptor bound protein 2; TGF, Tissue growth factor; PLC, phospholipase C; mTOR, mammalian target of rapamycin; GSK, Glycogen synthase kinase; TNF, tumor necrosis factor; Sirt: Sirtuin; MMP, Matrix metalloprotease; ERK, extracellular-signal-regulated kinase; WNK, lysine deficient protein kinase 1; SPAK, SPS1-related proline/alanine-rich serine-threonine kinase.

AT_1_ R is a G protein-coupled receptor and is widely expressed in the heart, endothelium, smooth muscle, kidney (mainly in glomerulosa cells), brain, adipose tissue and adrenal glands (Li et al., 2012). AT_1_ R encourages intracellular pathways via activation of subunits of nicotinamide adenine dinucleotide phosphate (NADPH) oxidase, several protein kinases, transactivation of growth factor receptor, or direct interaction with AT_1_ R interacting proteins like Guanine nucleotide exchange factor (GEF)-like protein (GLP), AT_1_ R associated protein (AT_1_ R AP), phospholipase C (PLC γ1) and Janus activated kinase (JAK 2) (Figure 2). In addition, it also turns on several downstream signals, like mitogen-activated protein kinase/extracellular signal-regulated kinases (MAPK/ERK), Ras/Rho, and translocation of MAPK in the nucleus (Ahmadian et al., 2015). AT_1_ R of mouse and rat exists as Ang II type I subtype A receptor (AT_1A_ R) and Ang II type I subtype B receptor (AT_1B_ R), which have similar activation, ligand binding properties, and identical amino acid sequences, but differ in tissue transcriptional and distributive regulation. AT_1A_ R is widely expressed and regulate blood pressure. Thus, it is anticipated to be the closest homolog to the human AT_1_ R (Touyz and Berry, 2002).

### G-Protein, Protein Kinases, Nicotinamide Adenine Dinucleotide Phosphate , and Growth Factor-Mediated Signaling

Ang II activation of AT_1_ R promotes variously convoluted, convergent, and diverse signaling pathways. However, research has established specific components essential for Ang II dependent signaling pathways. In brief, AT_1_ R binds with heteromeric G-protein (G_q/11_, G_i_, G_12,_ and G_13_) and allows activation of secondary messengers such as Rho GEFs, PLCβ, inositol 1,4,5-trisphosphate (IP_3_), diacyl glycerol (DAG), and reactive oxygen species (ROS). This further regulates downstream effectors like phospholipases. The response may differ depending on effector tissue, such as in vascular smooth muscle cells (VSMCs) contraction is regulated through G_12/13_ Rho/Rho kinase-mediated myosin light chain phosphatase (MLCP) inhibition or G_q/11_ Ca^2+^sensitive myosin light chain kinase (MLCK) activation. Similarly, Src family kinase also regulates vascular contraction through MLCP inhibition and Rho kinase/RhoA signaling. Ang II mediated AT_1_ R potentiates various serine/threonine kinases such as PKC, Akt, and mitogen-activated protein kinase (MAPK) family kinases and other intracellular protein kinases like, non-receptor and receptor tyrosine kinases (Figure 2 and Table 2).

**TABLE 2:** Identified protein kinases in ANG II signaling in cardiovascular system.

S. No.	Kinase	Associated physiology	References
**Ser/Thr kinase**			
1.	ERK	Stimulate NADPH oxidase and ROS generation causing hypertrophy, hyperplasia, and migration of VSMCs	Moraes et al. (2017)
			Chen et al. (2020)
			Ge et al. (2021)
2.	JNK	Cardiac hypertrophy	Xu et al. (2021)
3.	P38	Cardiac hypertrophy	Xu et al. (2021)
4.	MAPK	Cardiac hypertrophy, hyperplasia and migration of VSMCs	Chen et al. (2020)
5.	GRK	Regulate function of GPCR	Rukavina Mikusic et al. (2020)
			Murga et al. (2019)
			Brinks and Eckhart (2010)
6.	ROCK	Fibrosis and involved in TGF-β1-induced atrial remodeling.	Brinks and Eckhart (2010)
			Bai et al. (2021); Liu et al. (2016)
7.	PAK1	Attenuation of cardiac fibrosis and hypertrophy	Zhou et al. (2021)
8.	Raf	Phosphorylates and activates the MAPK kinase, MEK-1, which, in turn, phosphorylates and activates MAPK.	Ge et al. (2021)
9.	MLCK	Regulating cardiac muscle contraction and hypertrophy	Wang et al. (2017)
10.	CaMKII	Regulates Erk1/2 and Akt-dependent signaling in VSMC	Li et al. (2010)
11.	IKK	Triggers myofibroblast survival	Xu et al. (2021)
			Cao et al. (2021)
12.	PI3K	Stimulate NADPH oxidase and ROS generation causing hypertrophy, hyperplasia and migration of VSMCs	Zhong et al. (2021)
			Cheng et al. (2021)
			Gao et al. (2021)
13.	P70S6K	Stimulate NADPH oxidase and ROS generation causing hypertrophy, hyperplasia and migration of VSMCs	Gao et al. (2021)
14.	Akt	Stimulate NADPH oxidase and ROS generation causing hypertrophy, hyperplasia and migration of VSMCs	Gao et al. (2021)
			Kim et al. (2012)
15.	mTOR	Cell proliferation, motility and protein synthesis	Kim et al. (2012)
16.	PERK	Inhibition of protein synthesis	Fang et al. (2020)
			Lins et al. (2021)
17	AMPK	Preventive in AAA, endothelial dysfunction	Chen et al. (2020)
18.	ALK1/2/4	Central regulation of hypertension, cardiac fibrosis, cardiac hypertrophy	González-Núñez et al. (2015)
19.	MNK	Attenuation of cardiac fibrosis and hypertrophy	Kasuya et al. (2021)
			Yuan et al. (2016)
20.	WNK	Hypertension and vascular contraction	Simoes et al. (2020)
			Shao et al. (2021)
			Brown et al. (2021)
21.	SPAK	Hypertension and vascular contraction	Shao et al. (2021)
			Brown et al. (2021)
22.	MKK4	Atrial fibrosis via kinase activation	Fang et al. (2020)
			Li et al. (2017)
23.	TRPM7	Implicated in cardiac fibrosis	Zhong et al. (2018)
24.	DAPK	Vascular constriction via kinase activation	Usui et al. (2012)
25.	SGK1	Cardiac remodelling	Ock et al. (2021)
26.	PKD1	Cardiac hypertrophy and fibrosis	Weber et al. (2004); An et al. (2020)
27.	PKC	Stimulate NADPH oxidase and ROS generation causing hypertrophy, hyperplasia and migration of VSMCs	Wang J. et al. (2020)
28.	PKA	Cardiac hypertrophy	Wang J. et al. (2020)
			Dai et al. (2019)
**Tyrosine kinase**			
1.	Axl	Inhibitor of innate immunity	Berk and Corson, (1997)
			Batchu et al. (2016)
2.	Src	Hypertension and hypertrophy	Callera et al. (2016)
			Luo et al. (2020)
			Touyz et al. (2002)
			Berk and Corson, (1997)
3.	BMX	Cardiac hypertrophy via endothelial activation	Holopainen et al. (2015)
			Mitchell-Jordan et al. (2008)
4.	sFLT-1	Anti-angiogenic	Berk and Corson, (1997)
5.	FAK	Enhance protein synthesis	Berk and Corson, (1997)
6.	PYK2	Allows growth-promoting signal by Ang II in VSMC	Gao J. et al. (2020)
			Jain et al. (2021)
7.	JAK	Mediates Ang II triggered gene transcription	Han et al. (2018)
8.	EGF	Cell hypertrophy and remodeling	Vukelic and Griendling, (2014)
			Peng et al. (2016)
9.	PDGF	Cardiac remodeling	Weber et al. (2004)
			Touyz et al. (2002)
10.	IGF	Cardiac remodeling and hypertension	Ock et al. (2021)
11.	FGF-2	Cardiac hypertrophy	Li et al. (2019)
			Liu et al. (2020)

Akt, Protein kinase B; ALK, activin receptor-like kinase; AMPK, AMP-activated protein kinase; BCR, breakpoint cluster region protein; CaMKII, calmodulin-dependent protein kinase II; DAPK, death-associated protein kinase; EGF, Epidermal growth factor; EKR, extracellular-signal-regulated kinase; FAK, Focal adhesion kinase; FGF-2, Fibroblast growth factor; GRK, G protein-coupled receptor kinase; IGF, Insulin like growth factor 1; IKK, IκB kinase, JAK, Janus kinase; JNK, c-Jun N-terminal kinase; MAPK, Mitogen activated protein kinase; MKK4, mitogen-activated protein kinase kinase 4; MLCK, Myosin light chain kinase; MNK1, mitogen-activated protein kinase-interacting kinase 1; mTOR, Mammalian target of rapamycin; P70S6K, Ribosomal protein S6 kinase beta-1 PAK1, p21-activated kinase 1; PDGF, Platelet derived growth factor; PERK, protein kinase R-like ER kinase; PI3K, Phosphatidylinositol 3-kinase; PK, Protein kinase; PKD1, protein kinase D1; ROCK, Rho-associated protein kinases; Raf, Rapidly accelerated fibrosarcoma; Sflt-1, Soluble fms-like tyrosine kinase-1; SGK1, serum-glucocorticoid regulated kinase 1; SPAK, STE20/SPS1-related proline/alanine-rich kxinase; TRPM7, transient receptor potential 7; WNK, with no lysine kinase.

Ang II stimulates nicotinamide adenine dinucleotide phosphate (NADPH) oxidases to produce ROS causing renal deterioration, cardiac hypertrophy, and VSMC migration. In general, Ang II increases the production of ROS via activation of the catalytic subunit of NADPH, the Nox family proteins. The catalytic subunits of NADPH include dual oxidase (Duox1 and Duox2) and the Nox family (Nox1-Nox5). Subsequent stimulation of Nox family proteins increases its interactions with associated specific regulatory subunits p67 phox, p47 phox, p22 phox, and Nox1 (Figure 2) (Kawai et al., 2017). Ang II stimulated Nox4 generation in vascular cells and renal tissues via AT_1_ R is a source of oxidative stress, hypertension and organ failure. Ang II upregulates JNK and Nox4 in BubR1 (budding uninhibited by benzimidazole-related 1) low-expression mice (Aoyagi et al., 2019). Moreover, it stimulates the expression of AT_1_ R and receptors for advanced glycation end products (RAGE) by stimulating the PKC-ERK-NF-κB signaling pathway. In addition, it can increase intracellular ROS and critical mediators of cardiomyocyte hypertrophy by regulating expression levels of NADPH oxidase 2/4 in H9c2 cells (Lee et al., 2020).

Ang II mediated transactivation of nonreceptor tyrosine kinase (Janus kinase, c-Src, and focal adhesion kinase) and tyrosine kinase (EGF, PDGF, IR, etc.) resulted in modulation of various downstream targets like MAPK (Vukelic and Griendling, 2014). It can act as a growth factor that can regulate cell hypertrophy in VSMCs. Investigations have established dynamic phenomenon for AT_1_ R dependent transactivation of growth factor receptor-like EGF receptor (EGFR), followed by subsequent activation of Akt/p70S6, mechanistic target of rapamycin (mTOR), and Ras/ERK signaling resulting in cardiac hypertrophy and fibrosis (Eguchi et al., 1999; Eguchi et al., 2001; Ohtsu et al., 2006). Ang II stimulates selective mTOR2-dependent phosphorylation of SGK1 but not Akt (Gleason et al., 2019). The transactivation is mediated by secondary messengers like ROS, PKC, Src kinase, and metalloproteinase-dependent release of EGFR ligands such heparin-binding EGF, TGF- α, and EGF. Peng et al., 2016 evidenced that c-Src dependent EGFR transactivation in ERK/Akt pathway may a play crucial role in Ang II induced cardiac remodeling in H9c2 cells (Peng et al., 2016). AT_1_ R mediated A Disintegrin And Metalloproteinase 17 (ADAM17) dependent EGFR activation results in VSMC migration and hypertrophy via PI3K/Akt/mTOR/p70S6K pathway and Ras/ERK pathway. Ang II mediated ADAM17 requires ROS and p38 MAPK phosphorylation. Zhang Y. et al. (2019) demonstrated that Ang II promotes Mer tyrosine kinase shedding via AT_1_R/ROS/p38 MAPK/ADAM17 pathway in macrophages of ApoE^−/−^ mice (Zhang Y. et al., 2019). Furthermore, BMX (bone marrow kinase), a non-receptor tyrosine kinase has been identified as an upstream signalling molecule for Ang II-mediated EGFR activation. Thus, systemic inhibition of EGFR or ADAM17 decreases Ang II-induced cell migration and aortic aneurysm.

Ang II-dependent connective tissue growth factors (CTGF) and transforming growth factor-β (TGF-β) are initial pro-fibrotic mediators involved in cardiac fibrosis (Wong et al., 2018; van Beusekom and Zimmering, 2019). The expression of CTGF and TGF-β are interlinked. Ang II increases mRNA expression of TGF-β and NF-κβ, an important mediator of the hypertrophic growth of the heart, in H9c2 cells (Prathapan et al., 2013). Further, myocardial CTGF expression after Ang II exposure is likely dependent on latent activation of TGF-β via canonical Smad-pathway in NIH/3T3 fibroblasts (Wong et al., 2018). Overexpression of fibroblast growth factor 23 (FGF23) augmented cardiac fibrosis and hypertrophy in Ang II administered mice via PPARα/PLCγ-NFAT1/TGF-β signaling (Liu et al., 2020). Contrarily, FGF21 enhances cardiac function and reduces Ang II induced cardiac hypertrophy through in silent information regulator 1 (SIRT1)/adenosine monophosphate-activated protein kinase (AMPK) pathway (Li S et al., 2019). In addition, Ang II is well known to transactivate insulin-like growth factor I (IGF-I) receptor (IGF-IR) and platelet-derived growth factor (PDGF) receptor (PDGFR) in VSMC (Du et al., 1996). However, unlike EGFR, research on the role of Ang II-mediated IGF-IR, TGF, and PDGFR in cardiovascular pathophysiology is still limited.

Ang II-induced signaling via AT_1_ R is correlated with MAPK activation and enhanced phosphorylation of protein tyrosine. This fact highlights that besides vasoconstriction, Ang II also possesses the inflammatory and mitogenic properties. Similar to AT_1_ R, the existence of AT_2_ R is also opting for increased attraction due to its opposite effect than the former. AT_2_ R also belongs to the GPCR family and stimulates the SH2 domain-containing phosphatase (SHP-1) and MAPK phophatase1 (MKP-1) resulting in attenuation of tyrosine phosphorylation. In addition, AT_2_ R accelerates vasorelaxation through PKA-dependent eNOS activation and paracrine signaling through bradykinin/cGMP/NO production. Considering AT_2_ R, some AT_2_ R interacting proteins have shown physiological roles in the suppression of tumors, inflammation, ROS production and hypertrophy.

### G-Protein Independent Signaling via β-arrestin

Ang II stimulated AT_1_ R can activate various signaling cascades such as G-protein independent and G-protein dependent signaling. Unlike G-protein dependent signaling, the G-protein independent signal transduction cascade includes G-protein and β-arrestin (Forrester et al., 2018). Isoforms of β-arrestin i. e., β-arrestin 1 and 2 are recruited to AT_1_ R and stabilize them with high-affinity conformations (Sanni et al., 2010). Mechanistically, β-arrestin were described as a protein that uncouples GPCR from G-protein for mediating receptor internalization and G-protein independent signaling. β-arrestin mediated signaling includes activation of p38 and Akt/MAPK, JUNK, ERK1/2, and Src tyrosine kinases. A study involving human embryonic kidney (HEK)-293 cells biased agonist Sar^1^, Ire^4^, Ile^8^-ANG II (SII) or a mutant AT_1_ R-DRY/AAY suggested various active conformation of AT_1_ R. The SII or mutant AT_1_ R induced G protein independent, but β-arrestin 2-dependent ERK activation (Wei et al., 2003).

AT_1_ R, both β-arrestin and G_q/11_ proteins mediate biased signaling (Ferraino et al., 2021). β-arrestin-biased AT_1_ R signaling promotes vascular remodeling with the activation of MAPK and Src-based signaling. Interestingly, mechanical activation of AT_1_ R caused increased affinity toward β-arrestin biased ligand TRV 120023, suggesting stabilization of a biased active receptor conformation (Ma et al., 2021). Apart from its involvement in β-arrestin-dependent signaling, AT_1_ R was also reported to be involved in stretch-induced pathways in different cells. Interestingly, GRK2 and PKC, the kinase responsible for β-arrestin binding of many GPCRs have also been found to be activated up on a stretch in rat ventricular myocytes (Turu et al., 2019). In a study, the vasoconstrictor responses were increased by G_q/11_ AT_1_ R biased agonists TRV120055 and TRV20056. Here, G_q/11_ AT_1_ R was an essential component of dynamic mechanochemical signaling in VSMC causing myogenic tone (Cui et al., 2020). Alongwith AT_1_ R, AT_2_ R is also suggested to be primarily stimulated via G-protein independent signal transduction cascade including β-arrestin and GPCR kinase.

## Angiotensin II in Cardiovascular System

As a vital bioactive peptide of RAS, Ang II is associated with diverse mechanistic insights into understanding how Ang II contributes to multiple cardiovascular physiology and pathophysiology functions. Century-old research on RAS has uncovered Ang II and its involvement in the pathophysiology of cardiovascular diseases. Ang II is involved in the regulation of cell communication, impulse propagation, cardiac contractility, apoptosis, growth, and remodeling (Kawai et al., 2017). Summary of *in vivo* and *in vitro* pharmacological investigations are presented in Figure 2 and Table 3. In most of the *in-vivo* studies, the approach used for induction was a subcutaneous infusion of Ang II.

**TABLE 3:** Studies of effect of angiotensin II in *in-vivo* and *in-vitro* studies.

Model	Dose, Route, Duration of Ang II	Result	Limitations	Ref.
Sprague-Dawley rats and cardiomyocytes	20 μM, 2 h	Short-term treatment with Ang II attenuates the transversal YM in isolated adult rat cardiomyocytes acting via an AT_1_ R	High sample indentation in direct contact mode or lack of selectivity or that makes it difficult to assess the sample–probe interaction	Swiatlowska et al. (2020)
			Long exposure time to high-intensity light affecting cell enzymatic reactions, difficulty in manufacturing instruments, time-consuming measurements	
C57BL/6J mice & Primary cardiomyocytes from C57BL/6J mice	2.5 mg/kg/day, s.c., 2 weeks. 100 nM, 24 h	Administration of Ang II increases the expression of miR-154-5p and cardiac remodeling concurrently. miR-154-5p interacts with 3′ UTR and inhibits arylsulfatase B to trigger cardiomyocyte apoptosis and hypertrophy associated with oxidative stress	The hypothesis of miR-154-5p promoting hypertrophy needs further testing in the near future	Wang Q. et al. (2019)

HEK293T, HEK293-AT1R, and HEK293T-SIN1−/− cells	200 nM	SGK1 activation occurs at a distinct subcellular compartment from that of Akt	The use of SIN1 and SGK1 overexpression since overexpression of these proteins might influence their subcellular localization.	Gleason et al. (2019)
ApoE^−/−^ mice	750 µg/kg/day, s. c.	Ang II increases the expression of EMMPRIN in atherosclerotic plaque	Further research is required to elucidate details of the mechanism involved	Zhang Y. et al. (2019)
Amniotic fluid mesenchymal stem cells	0.1 and 1 μM	Ang II and TGF-β1 are efficient cardiomyogenic inducers of human AF-MSCs; They initiate protein expression, alterations at the gene and epigenetic levels in stem cells leading towards cardiomyocyte-like phenotype formation.		Gasiūnienė et al. (2019)
Male silent information regulator 1 (SIRT1) flox/flox and cardiomyocyte-specific inducible SIRT1 knockout mice (SIRT1-iKO)	1.1 mg/kg/day for 4 weeks	FGF21 improves cardiac function and alleviates Ang II-induced cardiac hypertrophy in a SIRT1-dependent manner	Presence of a small number of animals in a group	Li et al. (2019)
CRFK cells (feline kidney epithelial cell line)	-	Ang II shows a similar result to TGF-β1 if the AT_1_ R was expressed more in CRFK cells	The experiment could have involved other cells.	van Beusekom and Zimmering, (2019)
C57BL/6J mice	1.5 μg/min/kg, s.c., 4 weeks	Soluble receptors for advanced glycation end-products were evidenced to attenuate Ang II-induced LV hypertrophy using a 9.4T pre-clinical magnetic resonance imaging instrument	Since they didn’t perform electrocardiography, they were unable to confirm the superiority of MRI in assessing cardiac remodeling	Gao Q. et al. (2020)
Thromboxane A2 (TP) knockout (Tp−/−) mice	1,000 ng/kg/min, s.c., 28 days	TP receptors may contribute to cardiac hypertrophy but not, proteinuria and are responsible for thepathogenesis of Ang II induced hypertension and hypertrophy	As thromboxane production was not analyzed in Cox1^−/−^ mice, they were unable to assure the reduction caused by TXA2	Heo et al. (2019)
Sprague-Dawley rats	200 ng/kg/min, micro-infusion	Ghrelin inhibited Ang II-induced cardiac fibrosisin a PPAR-dependent manner	The study was performed on young male rats which restricts the extrapolation of results for females and older cohorts.	Zhong et al. (2018)
			Also, the age and sex-mediated effects of ghrelin need to be explored.	
Rat tubular epithelial cell line NRK52E	1 mM for 0–24 h	Inhibition of HMGB1 and gene silencing of TLR4 decreases Ang II-mediated inflammation in the kidney	Future *in-vivo* studies will be required for elucidating the role of TLR4 signaling in Ang II-induced renal injury on the AT_1_ R knock out model	Nair et al. (2015)
		The existence of HMGB1-TLR4 signaling is a development of hypertensive renal injury		
Mouse Neuro-2a cells	-	Involvement of HMGB1 in the PVN for development of Ang II-induced hypertension	Further research depicting the involvement of Mas will be necessary	Nair and Philips, (2015)
Sprague-Dawley rats	120 ng/kg/min, s.c.,2 weeks	Activation of brain RAS and PPAR-γ to reduce central inflammation may be used as a strategy in the management of Ang II-induced hypertension	Studies need to be performed to evaluate the relative role of individual types of cell	Yu et al. (2015)
Sprague-Dawley rats	100 ng/kg, i. c. v., before and after a 1 h ICV infusion of inhibitor	Role of brain p44/42 MAPK signaling cascade in the maintenance of renal sympathetic excitation in HF rats.	They evaluated the involvement of p44/42 MAPK signaling in the brain containing presympathetic neurons of PVN neurons only and did not evidence the contribution of p44/42 MAPK signaling in other nuclei of brain-like RVLM or other neurons in cardiovascular and autonomic centers, including organum vasculosum of the lamina terminalis, median preoptic nucleus, and the subfornical organ.	Shinohara et al. (2015)
		Alteration in brain p44/42 MAPK can increase adverse effects of brain RAS on renal and cardiovascular functions during HF progression.		
		Attenuation in Fra-LI–positive PVN neurons in p44/42 MAPK inhibitors treated rats suffering from HF.		
Sprague-Dawley rats	800 ng/kg/min, s.c., 1 week	Association of NO-mediated mechanisms with presence of female sex hormones to be protective against sympathetically mediated Ang II-induced hypertension in female mice	-	Wattanapitayakul et al. (2000)

### Angiotensin II in Hypertension

Hypertension is one of the most critical predisposing factors for the development of cardiovascular disease. Several factors contributing to the pathogenesis of hypertension include salt intake, stress, and Ang II (Kulkarni et al., 1998; Meneton et al., 2005). Ang II as a powerful vasoconstricting agent can induce aldosterone secretion and thereby retention of salt and water, which regulates blood pressure. In addition to this, Ang II has also been shown to have important oxidative, inflammatory, and immune-mediated actions. Ang II even at doses that do not alter blood pressure, allows the migration of inflammatory cells and induces the expression of inflammatory markers in the aorta of normotensive mice (Lima et al., 2019)

Nair *et al.* in 2015 found toll-like receptor 4 (TLR4), high-mobility group box 1 (HMGB1), and proinflammatory cytokines mediated immune response contributed to Ang II-induced hypertension in rat tubular epithelial cell line NRK52E (Nair et al., 2015). In support of this concept, they designed another study to determine the involvement of HMGB1 signaling in Ang II-induced hypertension in the para ventricular nucleus (PVN). The interaction between the inflammatory cytokine protein, HMGB1, and TLR4, resulted in the up-regulation of NF-kB which in turn resulted in hypertension in Neuro-2a cells of mice treated with Ang II (Nair and Philips, 2015). These findings were consistent with reports by Li et al. (2016) and Yu et al., 2015 that showed Ang II increases hypertension and hypothalamic infiltration via TLR4/MyD88/NF-κB signaling pathway and peroxisome proliferator-activated receptor-γ (PPAR-γ) in the PVN in hypertensive rats (Yu et al., 2015; Li et al., 2016).

In addition to, TLR4 and PPAR-γ, Ang II is known to induce ROS via activation of NADPH oxidase mediated Nox-1 and p22phox in Ang II-induced DNA damage *in-vivo* and *in-vitro* models (Wattanapitayakul et al., 2000; Sarr et al., 2006; Zimnol et al., 2020). Hyperactivation of Nox acts as a major source of ROS production in cardiac tissue, promotes apoptosis and increases oxidative stress via the MAPK pathway (Wen et al., 2019). Ogola et al., 2019 evidenced that pretreatment with G protein-coupled estrogen receptor (GPER) agonist G1 inhibits Ang II-induced ROS, NADPH, Nox4 mRNA expression via cAMP and phosphodiesterase inhibition (Ogola et al., 2019). Moreover, Ang II also induces pressor response and vasoconstriction by reduction of an active form of Ras-related C3 botulinum toxin substrate 1 (Rac1) and Nrf2 nuclear translocation (Pepe et al., 2019). Rac, a small G protein is an essential molecule for the function of NADPH oxidase components along with, phosphorylated Smad 2/3, atrial TGF-β1, and atrial superoxide in Ang II hypertensive rats (Yagi et al., 2010).

Another enzyme source of ROS includes uncoupled nitric oxide synthase (NOS), endoplasmic reticulum oxidase, xanthine oxidase, and mitochondrial oxidase. ROS affects the function of a cell by modifying proteins through post-translation modifications such as phosphorylation and oxidation (carbamylation, glutathionylation, nitrosylation, and sulfenylation). Proteins that are affected include matric metalloproteinases, cytoskeletal structural protein, transcriptional factors, signaling molecules, and ion transporter receptors (Griendling et al., 2016). ROS activate all 3 members of the MAPK family, including JNK, p38MAPK, and ERK1/2, essential for regulating vascular and cardiac cells (Jennings et al., 2010). In a study, catechins were reported to inhibit Ang II-induced VSMC proliferation by inhibiting Ang II activated MAPK and activator protein-1 signaling pathways (Won et al., 2006) (Figure 2). MEK-ERK are phosphorylated in arteries of hypertensive individuals and in a mouse model. Activation of this pathway results in the promotion of human arterial SMC (HASMCs). A known cysteine protease, Cathepsin L/V is interdependent on extracellular matrix accumulation and tissue inflammatory responses, allowing regulation of arterial remodeling. Lu et al., 2020 showed that Z-FF-FMK, a cathepsin inhibitor significantly reduces MEK-ERK phosphorylation (Lu et al., 2020).

Also, Ang II is responsible for causing atherosclerosis through VCAM 1 activation via protease-dependent NF-κB-like transcriptional mechanisms (Tummala et al., 1999). Acute doses of Ang II act primarily on VSMC to reduce blood pressure whereas chronic infusion of Ang II is neutrally mediated (∼10 h) (Li et al., 1996). It has been reported that statins reduce the incidence of cardiovascular remodeling. It is well known that apart from cholesterol-lowering, it also provides pleiotropic effects on the cardiovascular system, including anti-oxidant, anti-inflammatory, and improvement of endothelial function (Yagi et al., 2010). Candesartan, an AT1 R blocker and apocynin, NADPH oxidase inhibitor evidenced reduced pressor effect by AT1R-dependent ROS-SAPK/JNK, ERK1/2, and p38MAPK signaling (Jiang et al., 2019). Likewise, Pitavastatin exerts eNOS based protective action in Ang II-induced cardiovascular remodeling through suppression of (TGF)-β1–Smad 2/3 signaling pathway and oxidative stress (Yagi et al., 2010). Ang II inhibition extensively improved hypertension, hyperfiltration and control renal damage. Inhibition of Ang II enhanced the NF-KB activity which may additionally result in inhibition of its downstream gene expression, particularly NADPH-oxidase.

### Angiotensin II in Cardiac Remodeling

Cardiac remodeling can be described as a pathologic or physiologic condition that may occur after volume overload or idiopathic dilated cardiomyopathy, inflammatory heart muscle disease, pressure overload, or myocardial infarction (Cohn et al., 2000). Investigations have shown that Ang II promotes excessive accumulation of collagen leading to cardiac dysfunction as well as cardiac remodeling (Du et al., 2019). Several mechanisms have been implicated in the pathogenesis of Ang II-induced cardiac remodeling including dysfunction, hypertrophy, apoptosis and fibrosis. Ang II has been closely related to remodeling, which acts mainly via AT1 R in the animal and human cardiovascular systems. PKCs-EKR1/2-NFκB-NLRP3-IL1β pathway signaling cascades have been shown to promote Ang II-induced cardiomyocyte hypertrophy in H9c2 cells through AT1 R, RAGE, and NADPH oxidase inhibition (Lee et al., 2020). Soluble RAGE (sRAGE) was demonstrated as a decoy receptor for RAGE in Ang II-induced cardiomyocyte hypertrophy using *in vivo* and real-time 9.4T MR imaging (Heo et al., 2019). In addition to RAGE, it has been noted that Toll-like receptor 2 (TLR2)- and TLR4-dependent pathways are stimulated by Ang II in cardiac dysfunction, fibrosis and hypertrophy (Lee et al., 2020). TLR4 is involved in the upregulation of monocyte chemoattractant protein (MCP-1), IL-6, and ROS (Matsuda et al., 2015). Ang II stimulated direct binding of STAT3 with TLR4 activates STAT3 via IL-6/glycoprotein 130/JAK 2pathway, resulting in altered gene regulation for cardiac remodeling (Han et al., 2018).

Similarly, TGF-β has been proposed to act in a paracrine/autocrine manner between fibroblast and cardiomyocytes to stimulate cardiac remodeling (Leask, 2010). Valsartan, angiotensin receptor blocker, or Stachydrine mediated inhibition of Ang II/AT1 R/TGF-β signaling is a pivotal mechanism of anti-hypertrophic and anti-fibrotic effect (Teekakirikul et al., 2010; Liu et al., 2019; Tashiro et al., 2020). Ang II-induced fibrosis is associated with altered expression of inflammation-related genes such as TGF-β, TNF-α, MCP-1, IL-6, and type 3 collagen (Azushima et al., 2019).

miRNA including miR-154, miR-155, miR-132, miR-21, miR-503, miR-214, miR-19a, and miR-410 are involved in promoting hypertrophy, fibrosis, apoptosis, and inflammation. On other hand, miR-16, miR-98, miR-30a, miR-133, miR-433 possess cardio-protective effects (Wang Q. et al., 2019; Song et al., 2019; Adamcova et al., 2021). Luciferase assay evidenced that miR-214 acts as a target for Long non-coding RNA (lncRNA) Plscr4 and ameliorated levels of miR-214 oppose the anti-hypertrophic effect of Plscr4 in Ang II treated cardiomyocytes via lncRNA Plscr4-miR-214-mitofusin (Mfn2) axis (Lv et al., 2018). Ang II down-regulated expression of Neuregulin-1 (NRG-1) as a member of the epidermal growth factor family via the circNRG-1/miR-193b-5p-mediated post-transcriptional mechanism in mouse aortic smooth muscle cells (MASMCs) (Sun et al., 2019). Interestingly, the antiaging gene klotho modifies Ang II-induced cardiac remodeling via altering the expression of TGF-β and miR-132, a downstream mediator of TGF-β. LY364947, a TGF-β and klotho gene inhibitor, inhibited fibrosis, hypertrophy, expression of fibrotic marker genes (α-SMA, collagen I), pro-hypertrophic genes (atrial natriuretic peptide (ANP), brain natriuretic peptide (BNP), β-myosin heavy chain (β-MHC)) and Smad2/3 phosphorylation in cultured cardiomyocytes, fibroblasts and heart tissue (Ding et al., 2019). Zheng et al. evidenced similar results that liraglutide, a glucagon-like peptide-1 (GLP-1) receptor agonist, reduced protein levels of Smad2/3/4, AT1 R, TGF-β, collagen I and III and upregulated Smad7 in Ang II-induced fibrosis in rats (Zheng et al., 2019). Transient receptor potential subfamily M member 7 (TRPM7) is also implicated in cardiac fibrosis. Ang II-induced expression of p-Smad and collagen synthesis is inhibited by depletion of TRPM7 in cultured cardiac fibroblasts in rat sick sinus syndrome model (Zhong H et al., 2018).

p38 and MAPK pathways also play a crucial role in extracellular matrix deposition and cell proliferation during hypertensive cardiovascular remodeling (Jing et al., 2011; Jiajing et al., 2020). ERK represents MAPK and is involved in the growth and proliferation of VSMC. However, p38 MAPK inhibitor, PI3K inhibitor, and the c-JNK inhibitor didnot affect arterial blood pressure or renal sympathetic nerve activity in heart failure (Shinohara et al., 2015) (Figure 2). LCZ696, an AT1 R blocker, attenuated the hypertrophic expression of ANP, βMHC, and TIMP2 in Ang II-induced remodeling in cardiomyocyte and collagen I, collagen III, and TGF-β in cardiac fibroblasts via ERK inhibition (Wang Y. et al., 2019). A potent angiogenesis inhibitor, endostatin reduced the levels of ANP and BNP in primary neonatal rat cardiomyocytes and Ang II-treated rats via the cAMP/PKA pathway (Dai et al., 2019). Similar results were observed by ghrelin, a gut peptide that decreased Ang II-induced cardiac fibrosis by inhibiting TGF-β1 and upregulating PPAR-γ in rats. Also, it inhibits smooth muscle cell proliferation stimulated by angiotensin II by preventing cAMP/PKA signaling (Wang et al., 2018).

Astonishingly, a recent study suggested that PUO4F2/Brn-3b transcription factor acts as a novel regulator of adaptive hypertrophic responses in the adult heart along with other markers such as, ANP/βMHC. Levels of Brn-3b mRNA and related proteins are increased in Ang II treated mouse hearts and foetal heart-derived H9c2 cells or primary cultures of neonatal rat ventricular myocytes with varied hypertrophic alterations. Alternate signaling pathways involved in activation of Brn-3b promoter include p42/p44 MAPK/ERK1/2 or calcineurin pathways (Mele et al., 2019; Cheng et al., 2020). Cardiac fibrosis is induced by the pro (renin) receptor, which could be more worsened by the involvement PRR-ERK1/2-NOX4 pathway and ROS during the development of alcoholic cardiomyopathy (Cao et al., 2019). Ang II-induced collagen accumulation in cardiac fibroblast stimulates oxidative stress, ROS and inflammation via NF-κB/MAPK and nuclear factor erythroid 2-related factor (Nrf2)/AMPK pathway (Su et al., 2018; Du et al., 2019). Fibrotic proteins including, matrix metalloproteinases, collagen, and TGF are overexpressed in presence of Ang II through Akt/PI3K and NF-κB/MAPK pathway (Wang L. et al., 2019).

Despite the growing interest in big data approaches, with the aim of studying the genetics of cardiovascular disease, fishes are becoming an increasingly popular choice to study associated genetic alterations. To clarify the putative actions of Ang II in cardiac remodeling, *Anguilla Anguilla* was selected. Immunoblotting and immunolocalization results suggested that Ang II downregulates both localization and expression of molecules affecting apoptosis and cell growth such as eNOS, heat shock protein-90, and c-kit (Imbrogno et al., 2010; Imbrogno et al., 2013; Filice et al., 2017). Another recent study demonstrated the effect of Ang II on morpho-functional remodeling in heart of *Danio rerio*. The findings were paralleled by the upregulation of AT_1_ R and AT_2_ R expressions. Moreover, a significant change in expression of cytochrome b-245β polypeptide protein, superoxide dismutase 1 soluble mRNAs, NFk-light polypeptide gene enhancer in B cell, and GATA binding protein, indicated cardiac remodeling (Filice et al., 2021).

### Angiotensin II in Stem Cell Therapy in Cardiovascular System

Recent investigations have endeavored to improve stem cell functionality, provide stem cells as a promising therapeutic candidate for tissue transplantations in the cardiovascular system. Evolving research has evidenced the influence of RAS on stem cell growth, function, and proliferation (Becher et al., 2011). Several studies have demonstrated the role of Ang II in the differentiation of progenitor cell/stem cell (Matsushita et al., 2006). The presence of AT_1_ R in differentiated, but not in undifferentiated cells, suggests the concept that Ang II can regulate differentiation of stem cells (Huang et al., 2007). In addition to influencing a different kind of stem cells, the RAS effect on cardiovascular-related stem cell transplantation has largely been evaluated regarding the intracellular pathways of Ang II.

Mesenchymal stem cells (MSCs) are of great significance, along with their various autocrine-paracrine effects on the cardiovascular system and immune system (Zhang et al., 2020). Local RAS component Ang II has been reported to be expressed in rat MSCs. Vascular endothelial growth factor (VEGF) has been recognized in an invasion of extracellular matrix, migration, proliferation and survival of MSCs. Pre-treatment with Ang II increases mRNA expression of VEGF in MSCs through Akt/ERK1/2 signaling pathway via AT_1_ R. Considering this, pre-treatment of MSCs with LY292002, an Akt inhibitor attenuates Ang II-induced expression of VEGF. Notably, the involvement of Ang II increases the expression/production of VEGF in MSC grafts and improves transplantation efficiency (Liu et al., 2014). Thus, an angiogenic function of Ang II stimulates cells in ischemic regions through VEGF-induced endothelial nitric oxide synthase (eNOS). Ang II induces cardiomyogenic differentiation of rat bone marrow MSCs more efficiently than TGF-β1. The autocrine TGF-β/Smad pathway makes the differentiation of adipose tissue-derived MSCs to SMCs. Also, it acts synergistically with VEGF to ameliorate the differentiation of bone marrow-derived MSCs into endothelial cells (Ikhapoh et al., 2015).

Cardiac hypertrophy is a phenotypic response of the heart associated with various disorders. The genetic factor is an important determinant of phenotypic expression in hypertrophy (Marian, 2008). Neuron-derived orphan receptor-1 (NOR-1) transgenesis upregulates key genes involved in cardiac hypertrophy (Myh7, encoding for β-myosin heavy chain (β-MHC)) and fibrosis (Loxl2, encoding for the ECM modifying enzyme, Loxl2) in Ang II-induced cardiomyocytes (Cañes et al., 2020). In another study, Ang II and TGF-β1 upregulated the expression of the structured cardiomyocytes genes such as *DES, TNNT2* and *MYH6* as well as main cardiac genes-markers like GATA4, TBX5, and NKX2-5*.* Also, an increased expression of cardiac ion channel genes is evidenced with Ang II and TGF-β1 in human amniotic fluid-derived MSCs (AF-MSCs). Ang II and TGF-β1 treated AF-MSCs showed an increase in connexin43 protein and Nkx2.5 protein in AF-MSCs (Gasiūnienė et al., 2019). MSC can be an effective route for refining cell-based therapy of angiogenesis, vascular stabilization, and endothelial cell survival (Yang et al., 2019). Besides, TGF-β secretion is associated with the MAPK/ERK pathway; and Ang II in this pathway interferes with TGF-β production.

Bone marrow is one of the major Ang II producing organs and participant in the regulation of immunity and hematopoiesis (Yamashita et al., 2020). Being an inducer of differentiation of MSC, Ang II at doses ranged 0.1 to 10 μM can regulate apoptosis. Ang II could increase mitochondrial ROS through the activation of Nox2. In a study, Ang II at the dose of 1 and 10 μM leads to apoptosis in bone marrow-derived MSCs due to mitochondrial ROS production and mitochondrial DNA leakage mediated via AT_1_ R. Treatment with AT_1_ R inhibitor, losartan, markedly inhibited the Ang II-induced apoptosis and mitochondrial ROS (Zhang F. et al., 2019). Ang II production by cleaving enzyme chymase is several times higher in bone marrow than in other tissues. In a study, flow cytometry results showed Ang II generated via a chymase-dependent pathway in bone marrow was 280 -fold higher than in the heart. CD68^+^ myeloid progenitor possesses higher chymase expression than CD68^−^ progenitor cell in bone marrow (Yamashita et al., 2020). Another study showed that AT_1a_ R was widely expressed by human bone marrow CD34^+^, CD38^−^ cells, and lymphocytes (Rodgers et al., 2000). Ang II, but not Ang (1–7), increased adhesion of MNCs or CD34^+^ cells to fibronectin via ACE2/Ang-(1–7)/Mas pathway (Singh et al., 2015). The reported pathway stimulates vasoprotective functions of CD34^+^ cells.

Ang II inhibits colony growth by myeloid progenitors in a dose-dependent manner via AT_1_ R (Jokubaitis et al., 2008). Depletion of myeloid cells reduced vascular expression of AT_1_ R, adhesion molecule and vascular accumulation of oxidative stress, endothelial dysfunction, and Nox2^+^CD45^+^ cells (Molitor et al., 2021). Bone marrow-derived fibroblast precursors express certain chemokine receptors, such as CCR2, CCR5, CCR7, and CXCR4. Treatment of wild- type mice with Ang II (1,500 ng/kg/min) caused accumulation of bone marrow-derived fibroblast precursor expressing CD45, CD34, hematopoietic markers, collagen I, and mesenchymal markers. Whereas, the produced effects were abolished in CCR2 deficient mice depicting its role in the pathogenesis of Ang II-induced cardiac fibrosis (Xu et al., 2011). Everolimus, a rapamycin blocker, inhibited Ang II-induced aneurysm in ApoE^−/−^ mice through diminished M1 polarization and suppressed the development of bone marrow CCR2 monocytes (Moore et al., 2015). Ishibashi et al. evidenced that, MCP-1 regulates monocyte-mediated inflammation through leukocyte derived CCR2 receptor (C-C chemokine receptor) and its deficiency can reduce the pathogenic effect in Ang II (1.9 mg/kg per day, s. .c, 4 weeks) induced atherosclerosis and aneurysm in ApoE^−/−^ CCR2^−/−^ and ApoE^−/−^ CCR2^+/+^ mice (Ishibashi et al., 2004).

Autologous bone marrow MSCs are effective for regression of aneurysms in Ang II-induced ApoE^−/−^ mice (Akita et al., 2019). Bone marrow MSCs derived conditioned medium could prevent aneurysm growth through macrophage polarization regulation (Zhou et al., 2019). In addition, precursor fibronectin type III domain-containing protein 5 or irisin, a novel myokine has the potential to improve bone marrow MSC mediated paracrine effect and engraftments in infarcted hearts (Deng et al., 2020). Previous studies have shown that MSC-derived exosomes have distinct properties, including immunomodulation, angiogenesis, and paracrine effect that protect organ functions in animal studies. Additionally, recent investigations have evidenced that adipose-derived MSCs (ADMSCs) derived exosomes also possess a capacity of immunomodulatory, cardioprotective, and anti-inflammatory effects. Interestingly, irbesartan, an AT1 R blocker, was shown to abolish the effects of ADMSC-derived cell sheets in a rat model (Yamamoto et al., 2018). Administration of miR-19a/19b (exo/miR-19a/19b) using bone marrow-derived MSCs to cardiac HL-1 cells significantly suppressed the apoptosis and fibrosis in infarcted hearts (Wang S. et al., 2020).

Human-induced pluripotent stem cells (iPSCs) possess unique features to differentiate and self-renew into different types of cells in the body (Johansson et al., 2020). They are artificially derived from adult differentiated non-pluripotent somatic cells. In particular, these human cells-based models are anticipated to become an alternative for animal models. iPSCs can express Ang II receptors. As evidenced, Ang II induces the proliferation of PSC and allows their differentiation into MSCs. Treatment of PSC with Tempol, a ROS inhibitor, and Ang II reduces the cell proliferation and DNA synthesis, indicating the involvement of ROS signaling (Ahmadian et al., 2015). The presence and activation of the JAK/STAT pathway play a crucial role in stem cell renewal. It causes p38 phosphorylation and eventually, results in the differentiation of iPSCs in the target cell. A study showed that Ang II could stimulate hypertrophy in human embryonic stem cells (hESC)- and iPSCs derived cardiomyocytes (Földes et al., 2011). However, no significant effect was observed in cell death after treatment of Ang II (200 nM) with hESC- and iPSCs derived cardiomyocytes (Nunes et al., 2017). Immunofluorescent study and RNA seq revealed the involvement of low AT_1_ R expression in iPSC cardiomyocytes. Long-term Ang II incubation up-regulates AT_2_ R expression to induce downstream apoptosis signaling in iPSC-derived cardiomyocytes (Gao J. et al., 2020). Whereas, short-term Ang II treatment reduces Young modulus in rat cardiomyocytes via AT_1_ R. Inhibition using Rho-kinase or TGF-β1 abolished this effect (Swiatlowska et al., 2020).

Erythropoiesis is a tightly regulated process reinforced by systematic hematopoietic progenitor cells and some cohort of multipotent hematopoietic stem cells (HSCs) at its apex. HSCs form the basis of widely practiced therapies, including bone marrow transplantation and stem cell therapies (Jokubaitis et al., 2008). Chronic Ang II infusion imparts important regulatory roles on HSC proliferation, differentiation, and engraftment at the level of bone marrow (Kim et al., 2016) Flow cytometry analysis has revealed the involvement of Ang II in the regulation of hematopoiesis in HSCs and granulocyte/monocyte progenitor cells but, not in megakaryocytes/erythroid progenitors when coculture with stromal S17 cells. (Costa et al., 2021). In addition, protein microsequencing has also reported an increase in Gr-1+/Mac-1+ cells, BB9 protein and decrease expression of Ki67+ in presence of Ang II (Jokubaitis et al., 2008; Costa et al., 2021).

## Conclusion and Future Prospects

In the last decades, there has been a noteworthy progression in the number of molecules targeting Ang II signaling pathways. Since the discovery of Ang II, it has been characterized to be involved in various cellular activities such as proliferation, contractility, apoptosis, dysfunctions, remodeling, etc. Dysregulated Ang II signaling is considered to induce various cardiovascular diseases involving hypertension, inflammation, myocardial infarction, atherosclerosis, fibrillation, ventricular dystrophies, etc. While many ARBs are available commercially, the statistics indicate the need to look for novel mechanisms of Ang II causing cardiovascular diseases independently, which would provide a therapeutic target. The involvement of the RAS component in cardiovascular tissue and progenitor cells may regulate development and growth; and thus, allow the preparation of cardiac progenitor cells for clinical transplantation. Being a multi-functional peptide, Ang II stands amidst varied systems integrating multiple cellular signaling events that broadly have counterregulatory or opposing actions. The turmoil in the balance of such processes can lead to diverse pathologies and dysfunctions. Understanding how these processes are integrated with each other in real-time and remain a formidable challenge. Further studies with transgenics or cell-specific knockouts are needed to unravel the role of inflammatory and hypertrophic markers in Ang II-induced remodeling. Another challenge is providing evidence for working with cardiovascular progenitor cells for clinical transplantation. Further, the role of epigenetic/genetic programming impacting the cardiovascular system is an emerging area of study. Many questions still remain as well regarding paracrine, autocrine, and intracrine actions of Ang II and its interaction with receptor-associated proteins. In addition, an approach that integrates elements of metabolomics, proteomics, epigenomics, and genomics is likely to reveal novel, as yet unforeseen facet of Ang II signal transduction. Understanding diverse regulatory signaling mechanisms of Ang II can, therefore, offer better insight into various pathological states which in turn may help in designing ideal drug candidates for their management.

## Glossary

AA: Aortic aneurysm
ACE: angiotensin-converting enzyme
ApoE: apolipoprotein E
AR: angiotensin receptor
Ang II: Angiotensin II
AT_1_ R: Angiotensin type I receptor
AT_1_ R AP: AT I receptor-associated protein
AT_1A_ R: Ang II type I subtype A receptor
AT_1B_ R: Ang II type I subtype A receptor
AT_2_ R: Angiotensin type II receptor
AT_1_ R-(PI3K)/Akt: Ang II-mediated AT_1_ R- phosphoinositide 3-kinase/protein kinase B
CaN: calcineurin
CTGF: connective tissue growth factor
DAG: diacylglycerol
ECM: extracellular matrix
EGF: Endothelial growth factor
EGFR: Endothelial growth factor receptor
ERK: extracellular-regulated kinase
GLP-1: Glucagon-like peptide-1
HSC: hepatic stellate cells
HMGB1: high-mobility group box 1
ICAM: Intercellular Adhesion Molecule
IL: interleukin
iNOS: inducible nitric oxide synthase
IP_3_: inositol triphosphate
IRAK-4: Interleukin-1 receptor-associated kinase 4
JAK: Janus activated kinase
LVH: Left ventricular hypertrophy
miR: microRNA
MLCK: myosin light chain kinase
MLCP: myosin light chain phosphatase
MKP-1: MAPK phophatase1
MMP: mitochondrial membrane potential
MyD88: Myeloid differentiation factor 88
NADPH: nicotinamide adenine dinucleotide phosphate
NO: nitric oxide
PI3K: phosphatidylinositol 3-kinase
PLC γ1: phospholipase C
PLD: phospholipase D
PVN: para ventricular nucleus
RAS: renin angiotensin system
RASMC: rat aortic smooth muscle cells
ROS: reactive oxygen species
SFK: Src family kinases
SIRT1: sirtuin 1
SOD: superoxide dismutase
SPAK: Ste20/SPS1-related proline/alanine-rich kinase
SRA: suprarenal aorta
STAT: signal transducer and activators of transcription
TGF-β1: Transforming growth factor-β1
TLR4: Toll-like receptor 4
TRAF-6: TNF receptor-associated factor 6
TRPC6: transient receptor potential cation channel, subfamily C
VCAM: vascular cell adhesion molecule
VEGF: vascular endothelial growth factor
WNK: with-no-lysine
